# Plasma lipidomics profiling in predicting the chemo-immunotherapy response in advanced non-small cell lung cancer

**DOI:** 10.3389/fonc.2024.1348164

**Published:** 2024-07-08

**Authors:** Hui Jiang, Xu-Shuo Li, Ying Yang, Rui-Xue Qi

**Affiliations:** ^1^ Department of Ultrasound, Jinshan Hospital, Fudan University, Shanghai, China; ^2^ Department of Center for Tumor Diagnosis & Therapy, Jinshan Hospital, Fudan University, Shanghai, China

**Keywords:** chemo-immunotherapy, non-small cell lung cancer, lipidomics, biomarker, LC-MS

## Abstract

**Background:**

Advanced non-small cell lung cancer (NSCLC) presents significant treatment challenges, with chemo-immunotherapy emerging as a promising approach. This study explores the potential of lipidomic biomarkers to predict responses to chemo-immunotherapy in advanced non-small cell lung cancer (NSCLC) patients.

**Methods:**

A prospective analysis was conducted on 68 NSCLC patients undergoing chemo-immunotherapy, divided into disease control (DC) and progressive disease (PD) groups based on treatment response. Pre-treatment serum samples were subjected to lipidomic profiling using liquid chromatography-mass spectrometry (LC-MS). Key predictive lipids (biomarkers) were identified through projection to latent structures discriminant analysis. A biomarker combined model and a clinical combined model were developed to enhance the prediction accuracy. The predictive performances of the clinical combined model in different histological subtypes were also performed.

**Results:**

Six lipids were identified as the key lipids. The expression levels of PC(16:0/18:2), PC(16:0/18:1), PC(16:0/18:0), CE(20:1), and PC(14:0/18:1) were significantly up-regulated. While the expression level of TAG56:7-FA18:2 was significantly down-regulated. The biomarker combined model demonstrated a receiver operating characteristic (ROC) curve of 0.85 (95% CI: 0.75–0.95) in differentiating the PD from the DC. The clinical combined model exhibited an AUC of 0.87 (95% CI: 0.79–0.96) in differentiating the PD from the DC. The clinical combined model demonstrated good discriminability in DC and PD patients in different histological subtypes with the AUC of 0.78 (95% CI: 0.62–0.96), 0.79 (95% CI: 0.64–0.94), and 0.86 (95% CI: 0.52–1.00) in squamous cell carcinoma, large cell carcinoma, and adenocarcinoma subtype, respectively. Pathway analysis revealed the metabolisms of linoleic acid, alpha-linolenic acid, glycerolipid, arachidonic acid, glycerophospholipid, and steroid were implicated in the chemo-immunotherapy response in advanced NSCLC.

**Conclusion:**

Lipidomic profiling presents a highly accurate method for predicting responses to chemo-immunotherapy in patients with advanced NSCLC, offering a potential avenue for personalized treatment strategies.

## 1 Introduction

Lung cancer is the leading cause of cancer-related mortality globally, characterized by the highest incidence rates and a five-year survival rate of less than 20% (1). Non-small cell lung cancer (NSCLC) represents the majority, comprising 85%-90% of all lung cancer cases (2). NSCLC is associated with a diverse array of risk factors, from genetic mutations to environmental exposures, highlighting the need for sophisticated treatment modalities. Traditional therapeutic approaches have encompassed surgery, radiotherapy, chemotherapy, targeted therapy, and recently, immunotherapy (3).

Immunotherapy has notably advanced the treatment landscape for NSCLC, especially in the absence of targetable genetic mutations, offering a novel precision treatment modality (4). However, its effectiveness as a standalone treatment in advanced stages has been modest, benefiting only 15–20% of patients (5). The integration of chemotherapy with immunotherapy has generated optimism in the oncology community, demonstrating enhanced therapeutic outcomes in recent clinical trials (6). This combination therapy represents a pivotal shift in NSCLC treatment paradigms, offering hope for improved survival rates.

The role of biomarkers in predicting treatment response has become a focal point in NSCLC research. Biomarkers like PD-L1 expression (7), DNA methylation, tumor mutational burden (8), and T cell proliferation (9) have been instrumental in guiding treatment decisions. Nonetheless, the variable predictive value of these biomarkers underscores an imperative for novel, more reliable indicators (10, 11). This need is particularly pronounced in the context of chemo-immunotherapy, where patient selection is critical for maximizing therapeutic efficacy.

In the search for novel biomarkers, lipidomics presents a promising avenue. Lipids, essential in energy storage, signal transduction, and cell membrane formation, play significant roles in various diseases, including cancers (12). Particularly, Phosphatidylcholine (PC) constitutes about half of the phospholipids in cell membranes, playing key roles in maintaining their structure and function. Cholesteryl esters (CE) are crucial for cholesterol transport and storage. Meanwhile, fatty acids (FA) and triacylglycerol (TAG) can influence cancer cell proliferation (12, 13). The field of lipidomics, using advanced techniques like mass spectrometry, offers a comprehensive analysis of lipid profiles, potentially revealing disease-specific patterns. Mass spectrometry, recognized for its precision and evolving methodologies, has become a cornerstone in lipidomics research (13).

The application of lipidomics in NSCLC, particularly for predicting chemo-immunotherapy responses, is yet to be fully explored. This study aims to fill this gap by hypothesizing that lipidomic profiles could serve as effective biomarkers for predicting responses to chemo-immunotherapy in advanced NSCLC. Utilizing liquid chromatography-mass spectrometry (LC-MS), we analyzed the lipidomic profiles of advanced NSCLC patients. Our objective was to identify potential lipidomic biomarkers that could aid in predicting the efficacy of chemo-immunotherapy, thereby contributing to personalized treatment approaches and improved patient outcomes in advanced NSCLC.

## 2 Materials and methods

### 2.1 Ethics

This study was reviewed and approved by the Institutional Review Board of Jinshan Hospital (JIEC 2023-S84). Written informed consent was obtained from all participants. The methods were executed in accordance with relevant guidelines and regulations.

### 2.2 Study design and patient selection

Between October 2021 and October 2022, 76 patients were initially considered. Inclusion criteria were: (1) patients with stage IV NSCLC, (2) the treatment regimen included 4–6 cycles of cisplatin-paclitaxel combined with toripalimab, followed by toripalimab monotherapy. Exclusion criteria were: (1) presence of infection or inflammation symptoms, (2) patients undergoing radiotherapy or targeted therapy, (3) patients loss to follow-up. The study’s endpoint was six months post the initial chemo-immunotherapy cycle. Ultimately, 68 patients were included and classified into a disease control (DC) group (including complete response [CR], partial response [PR], and stable disease [SD]) and a progressive disease (PD) group. Tumor responses were evaluated using the Response Evaluation Criteria in Solid Tumors, version 1.1 (RECIST v1.1) (14).

### 2.3 Clinical data collection

Clinical data included age, gender, primary tumor and metastases location (lung, brain, liver and bone), and pathological subtype were collected. Multivariate logistic regression analysis was performed to select clinical independent predictors for responders of chemo-immunotherapy. Fasting peripheral blood (2 mL) was drawn within a week before the initial chemo-immunotherapy cycle using a serum separator tube.

### 2.4 Sample preparation and lipidomics analysis

Blood samples were centrifuged at 1,200 g for 10 minutes at 4°C within 30 min of collection. Plasma (20 μL) was mixed with 350 μL pre-cooled isopropanol and 9 μL of an internal standard mixture. After incubation at room temperature for 10 min and overnight storage at -20°C, the samples were centrifuged at 12,000 g for 20 min. The supernatant (200 μL) was stored at -80°C for subsequent LC-MS analysis.

Lipidomic profiling utilized an AB SCIEX QTRAP 5500 LC-MS system. Analytes were separated on a Waters Acquity UPLC BEH HILIC column, using a binary solvent system with specific acetonitrile-water ratios and ammonium acetate. The flow rate was set at 0.5 mL/min, and a gradient elution was performed. The analysis employed both positive and negative electrospray ionization (ESI) modes.

### 2.5 Lipid quantification and data analysis

Data were processed using Analyst (version 1.7) and MultiQuant software. The LIPID MAPS database was referenced for lipid identification and quantification. Lipids detected in at least 80% of samples in either group were included, with missing values replaced by the median. Data normalization was performed using Pareto scaling.

### 2.6 Lipidomic profile and key lipids identification

Principal component analysis (PCA) was first used to assess clustering patterns and outliers. Projection to latent structures discriminant analysis (PLS-DA) was then used to identify the key lipids based on variable influence on projection (VIP) scores, false discovery rates (FDR), and area under the receiver operator characteristic (ROC) curves.

### 2.7 Discrimination performance of key lipids and validation

The AUCs of the key lipids in differentiating PD and DC groups was reported. A biomarker combined model using binary logistic regression was constructed to enhance the discrimination efficiency between PD and DC groups. The combination of the biomarkers and clinical independent predictors (clinical combined model) was performed using binary logistic regression in differentiating PD and DC groups. The predictive performances of the clinical combined model in different histological subtypes were also performed.

Fifty percent of the cases were randomly selected to form the internal validation cohort. The discrimination performance of the biomarker combined model and the clinical combined model were evaluated.

### 2.8 Pathway analysis

The key lipids were integrated into the Kyoto Encyclopedia of Genes and Genomes (KEGG) database for pathway enrichment analysis via MetaboAnalyst.

### 2.9 Statistical analyses

Statistical analyses were conducted in R (Version 4.0.2). The PD and DC groups’ ages were expressed as mean (standard deviation) and compared using t-tests; gender, tumor and metastases locations, and pathological subtypes were expressed as N (%) and analyzed using Chi-Square tests. A p-value < 0.05 was deemed statistically significant.

### 2.10 Sample size calculation

We aimed to detect significant differences in lipid expression levels with adequate statistical power. The lipid expression levels were used as the primary outcome measure and assumed a moderate effect size (Cohen’s d = 0.5). We set a desired statistical power of 0.80 (80%) to minimize the risk of Type II error and a significance level of 0.05 was chosen to control the Type I error rate. Therefore, a sample size of approximately 34 participants per group would provide sufficient power to detect significant differences in lipidomic profiles between PD and DC groups.

## 3 Results

### 3.1 Patient demographics and baseline characteristics

**Figure 1:**
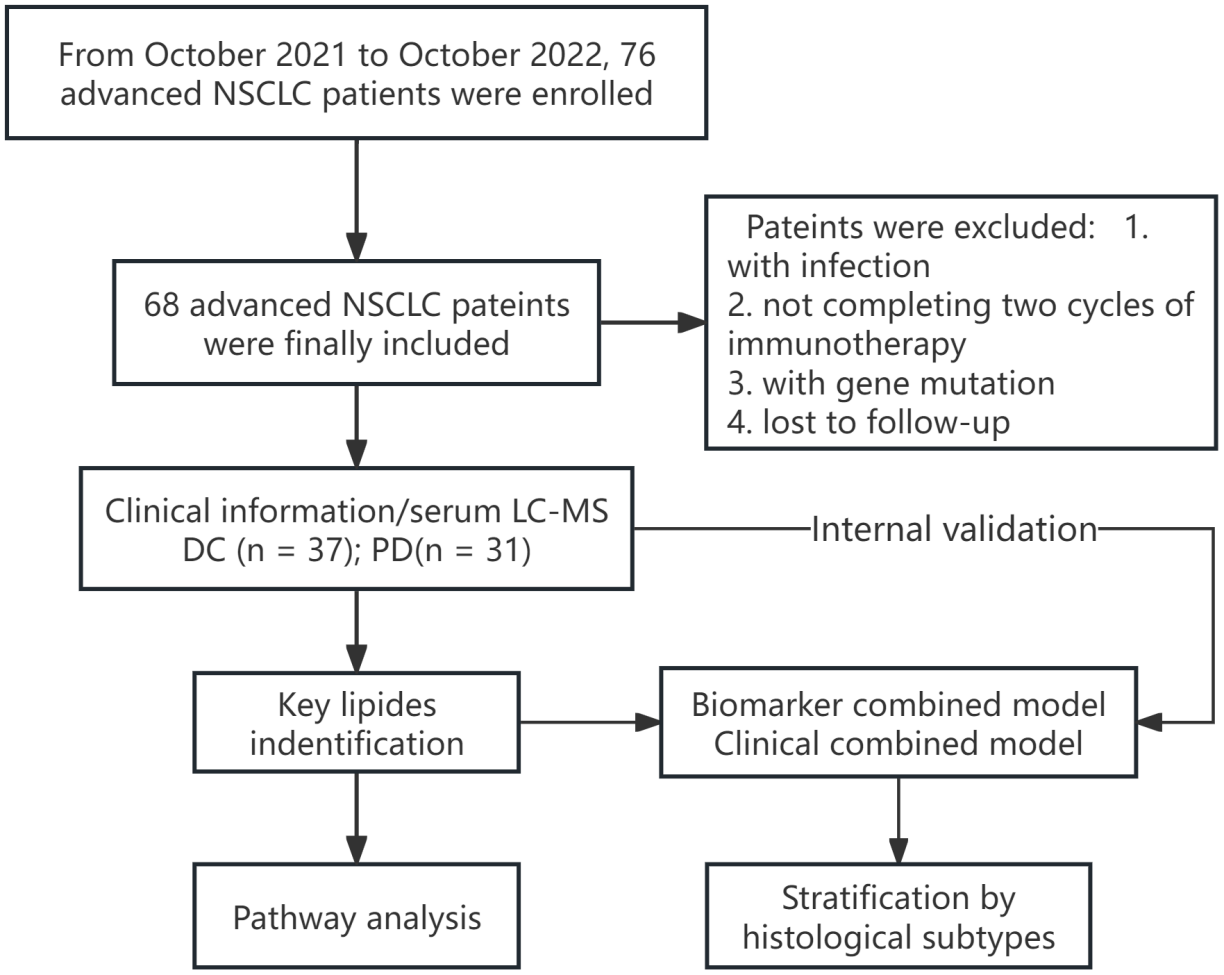
The work flow of this study. DC, disease control; PD, progressive disease.

The work flow of this study is shown in **Figure 1**. The study evaluated 68 patients with an average age of 64 (SD: 10.1), ranging from 44 to 86 years. The DC group included 37 patients, averaging 61 years (SD: 9.7), with a spread from 44 to 83 years. This group included 1 patient (2.9%) achieving CR, 2 (5.4%) with PR, and 34 (91.9%) with SD. It consisted of 11 females (average age 61, range 49–72) and 26 males (average age 60, range 44–83). The PD group comprised 31 patients, with an average age of 68 years (SD: 9.2), ranging from 51 to 86 years, including 9 females (average age 69, range 53–83) and 22 males (average age 67, range 51–86).

### 3.2 Clinical characteristics

No significant differences were observed between the DC and PD groups concerning gender distribution, tumor location, metastases sites (lung, brain, bone, and other sites) or pathological subtypes. However, patients in the PD group were older and exhibited more frequent liver metastases compared to the DC group (**Table 1**). Multivariate logistic regression analysis showed that liver metastases was the independently significant clinical predictor for responders to chemo-immunotherapy in advanced NSCLC (**Supplementary Table 1**).

**Table 1:** Comparison of clinical characteristics between DC and PD group.

	DC (N=37)	PD (N=31)	P
Endpoint			<0.001
SD	34 (91.9%)	0 (0%)	
CR	1 (2.7%)	0 (0%)	
PR	2 (5.4%)	0 (0%)	
PD	0 (0%)	31 (100%)	
Gender			1.000
Female	11 (29.7%)	9 (29.0%)	
Male	26 (70.3%)	22 (71.0%)	
Age	60.7 (9.73)	68.0 (9.20)	0.002
Tumor location			0.843
Central	9 (24.3%)	6 (19.4%)	
Peripheral	28 (75.7%)	25 (80.6%)	
Lung metastases			0.460
Negative	28 (75.7%)	20 (64.5%)	
Positive	9 (24.3%)	11 (35.5%)	
Brain metastases			0.346
Negative	25 (67.6%)	25 (80.6%)	
Positive	12 (32.4%)	6 (19.4%)	
Bone metastases			0.203
Negative	29 (78.4%)	19 (61.3%)	
Positive	8 (21.6%)	12 (38.7%)	
Liver metastases			0.003
Negative	33 (89.2%)	17 (54.8%)	
Positive	4 (10.8%)	14 (45.2%)	
Other metastases			1.000
Negative	35 (94.6%)	29 (93.5%)	
Positive	2 (5.4%)	2 (6.5%)	
Pathological subtype			0.114
Adenocarcinoma	23 (62.2%)	16 (51.6%)	
Large cell	2 (5.4%)	7 (22.6%)	
Squamous carcinoma	12 (32.4%)	8 (25.8%)	
Smoke			1.000
Negative	3 (8.1%)	3 (9.7%)	
Positive	34 (91.9%)	28 (90.3%)	
ECOG performance status	1.9 (0.6)	1.9 (0.4)	0.394

DC, disease control; PD, progressive disease. Data presented as mean (SD) or N (ratio).

### 3.3 Lipidomic profile and key lipids identification

**Figure 2:**
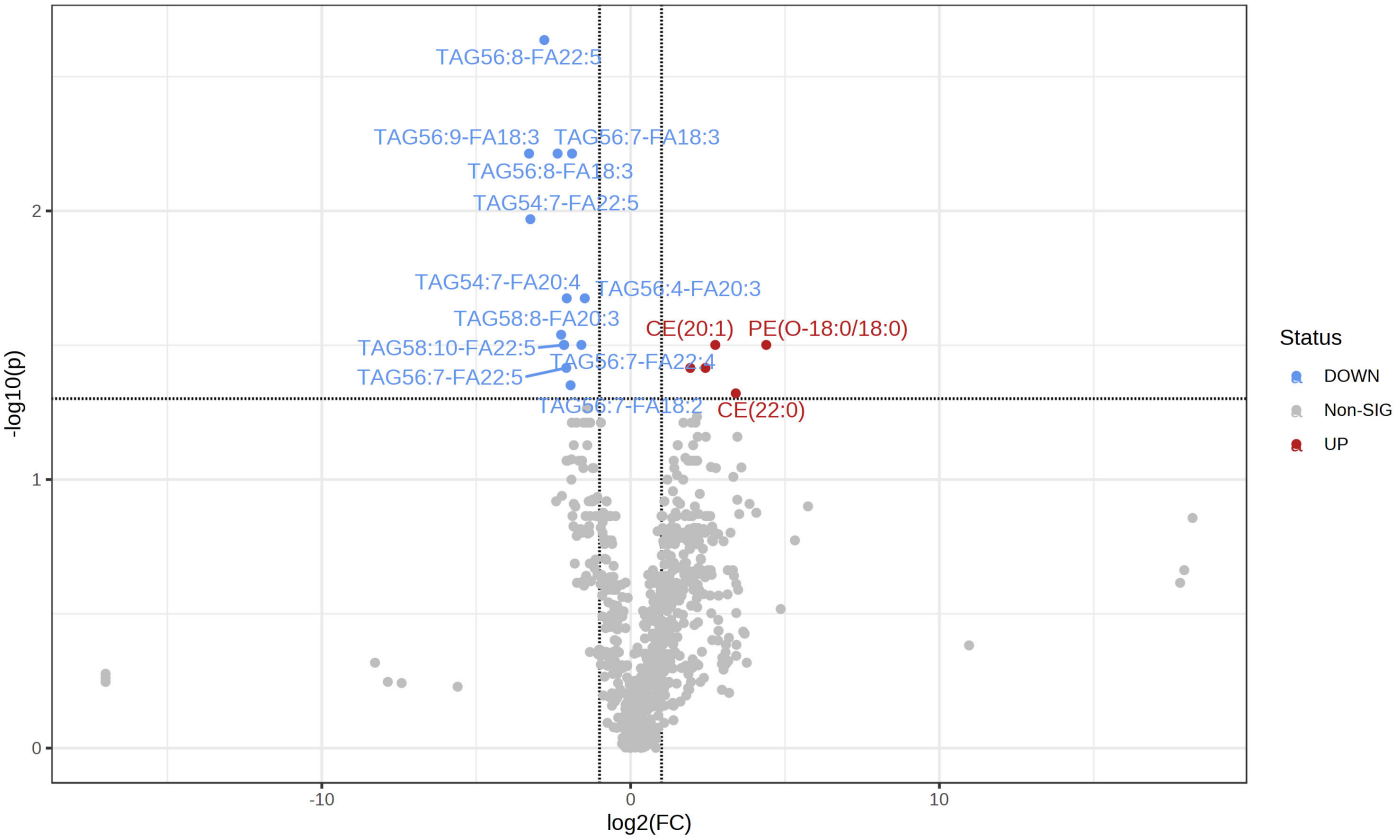
The volcano map shows the comparison of serum lipid content between the two groups (PD/DC). The decrease in lipid content is represented by blue, and the increase in lipid content is represented by red (FDR <0. 05).

**Figure 3:**
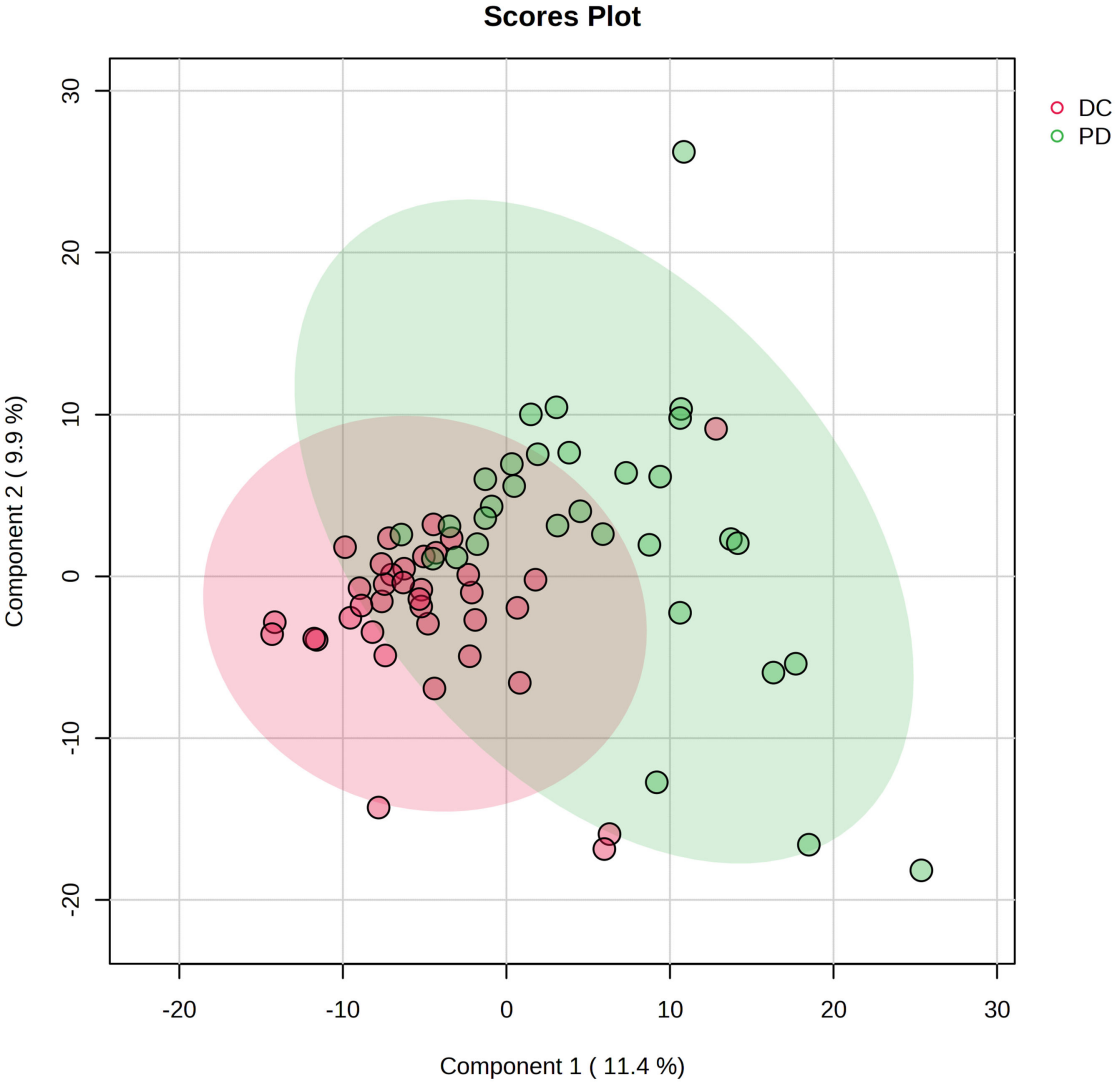
Projection to latent structures discriminant analysis (PLS-DA) of the lipids in DC and PD groups. The plot displays the distribution of advanced NSCLC patients based on their serum lipidomic profiles. The plot identifies two distinct clusters corresponding to patient responses to chemo-immunotherapy: DC (red circles) and PD (green circles). Each point represents an individual patient’s lipidomic profile projected onto the plane defined by the first two principal components, which capture the largest variance within the dataset.

**Figure 4:**
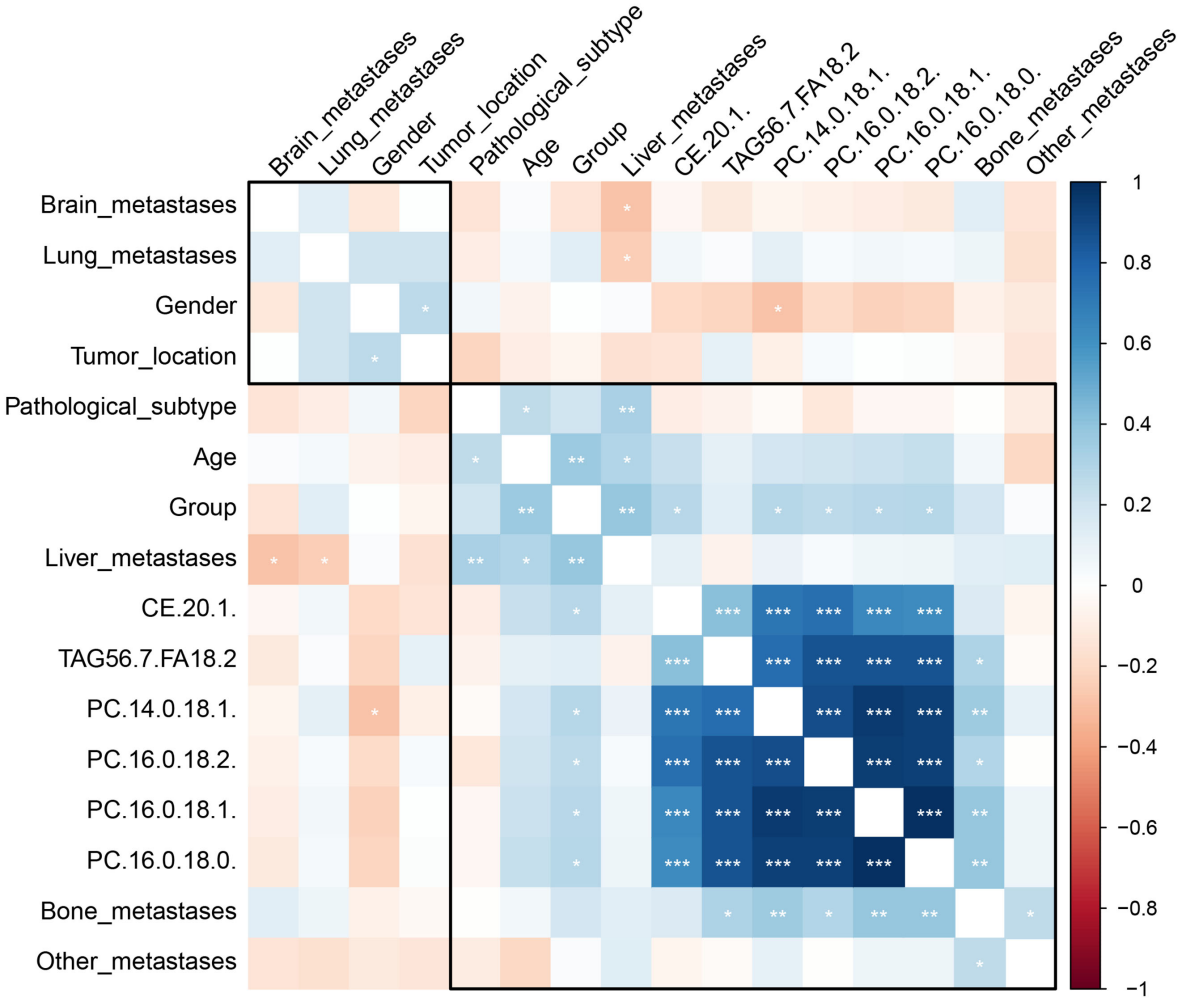
The heatmap depicts the correlation between clinical characteristics, patient demographics, a treatment group, and identified lipid biomarkers. The color gradient from blue to red represents the correlation coefficient values, with blue indicating a negative correlation (with DC), red a positive correlation (with DC), and the intensity of the color indicating the strength of the correlation. The stars represent the statistical significance of the correlation, with more stars indicating higher significance levels. *P < 0.05; **P < 0.01, ***P <0.001.

A total of 781 lipids were identified and quantified. PCA (**Supplementary Figure 1**) and volcano plots indicated differential lipid regulation between groups (**Figure 2**). PLS-DA effectively separated the lipid profiles of the DC and PD groups, with an accuracy of 0.71, R^2^ of 0.31, and Q^2^ of 0.07 (**Figure 3**). Six key lipids were identified with VIP > 1, FDR < 0.01, and AUC > 0.6, showing significant concentration differences between groups. A heatmap illustrated the correlation between these lipids and clinical characteristics (**Figure 4**). Six lipids were identified as the key lipids. The expression levels of Phosphatidylcholine (PC) including PC(16:0/18:2), PC(16:0/18:1), PC(16:0/18:0), and PC(14:0/18:1) and Cholesteryl Ester (CE) including CE(20:1) were significantly up-regulated. While the expression level of Triacylglycerol (TAG) and Fatty Acid (FA) includingTAG56:7-FA18:2 was significantly down-regulated.

### 3.4 Discrimination performance of key lipids and validation

The six key lipids showed varying abilities to discriminate to distinguish PD from DC, with AUCs spanning 0.60 to 0.75 (**Table 2**). A biomarker combined model (includes all six identified key lipids) was created using binary logistic regression, exhibited an AUC of 0.85 (95% CI: 0.75–0.95), with specificity, sensitivity, negative predictive value, and positive predictive value of 0.78, 0.87, 0.88, and 0.77, respectively, in differentiating the PD from the DC. The clinical combined model exhibited an AUC of 0.87 (95% CI: 0.79–0.96), with specificity, sensitivity, negative predictive value, and positive predictive value of 0.71, 0.94, 0.93, and 0.73, respectively, in differentiating the PD from the DC. There was no statistic significant between the AUC of the clinical combined model and the biomarker combined model (P = 0.590).

**Table 2:** The variable influence on projection score, false discovery rate, fold change, and area under the receiver operator characteristic curve of the six key lipids.

Lipids	VIP	FDR	FC	AUC	95% CI
PC(16:0/18:2)	11.6	0.003	1.52	0.71	0.58–0.83
PC(16:0/18:1)	8.91	0.002	1.56	0.61	0.50–0.75
PC(16:0/18:0)	1.61	0.003	1.36	0.65	0.52–0.79
CE(20:1)	1.38	0.001	3.46	0.75	0.63–0.86
PC(14:0/18:1)	1.08	0.004	1.35	0.61	0.47–0.76
TAG56:7-FA18:2	1.04	0.001	0.73	0.60	0.46–0.75

AUC, area under the receiver operator characteristic curve; FC, fold change (DC/PD); FDR, false discovery rate; VIP, variable influence on projection score. For the lipids, the first number (before the colon) indicates the number of carbon atoms in the fatty acid chain, while the number after the colon (): indicates the number of unsaturated bonds (double bonds). The slash (/) separates the two fatty acids, indicating that they are each attached to different positions on the glycerol backbone of the phospholipid.

The clinical combined model demonstrated good discriminability in DC and PD patients in different histological subtypes with the AUCs of 0.78 (95% CI: 0.62–0.96), 0.79 (95% CI: 0.64–0.94), and 0.86 (95% CI: 0.52–1.00) in squamous cell carcinoma, large cell carcinoma, and adenocarcinoma subtype, respectively.

Thirty-four cases were randomly selected to form the internal validation cohort. The discrimination performance, specificity, sensitivity, negative predictive value, and positive predictive value were 0.78 (95% CI: 0.60–0.96), 0.88, 0.75, 0.80, and 0.86, respectively, for the biomarker combined model and 0.83 (95% CI: 0.68–0.97), 0.72, 0.88, 0.87, and 0.74, respectively, for the clinical combined model.

### 3.5 Pathway analysis

**Figure 5:**
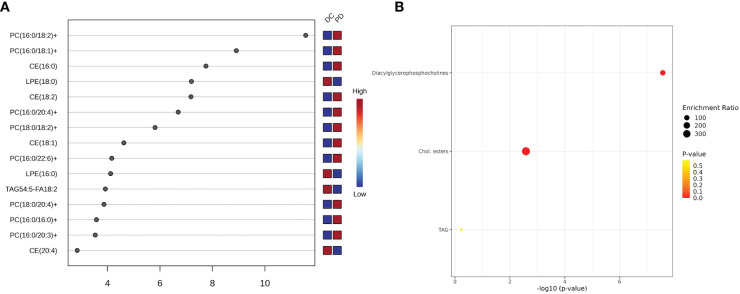
The key lipids and pathway analysis. **(A)** The variable influence on projection (VIP) score of the lipids by PLS-DA. Each dot represents a lipid species, and its position on the x-axis indicates the VIP score. The higher the score, the more influential the lipid is in distinguishing between the patient outcomes. The VIP scores suggest that certain lipid species, such as PC(16:0/18:2), is more predictive of patient response to chemo-immunotherapy. **(B)** Pathway analysis shows top three metabolism pathway involved in the chemo-immunotherapy treatment. The plot’s y-axis lists the metabolic pathways, while the x-axis indicates the significance of enrichment, which is a common transformation to highlight smaller p-values. The size of each bubble represents the enrichment ratio, and the color indicates the p-value, with warmer colors representing more statistically significant associations.

Pathway enrichment analysis identified several metabolic pathways involved in chemo-immunotherapy response, including linoleic acid, alpha-linolenic acid, glycerolipid, arachidonic acid, glycerophospholipid, and steroid metabolism (**Figure 5**).

## 4 Discussion

This research leveraged lipidomic analysis to identify biomarkers predictive of chemo-immunotherapy outcomes in advanced NSCLC. We pinpointed six key lipids with potential as prognostic indicators for treatment response, underscoring lipidomics’ utility in uncovering novel biomarkers and elucidating lipid metabolism’s role in cancer pathophysiology. A biomarker combined model (combination of the six key lipids) and a clinical combined model (combination of the six key lipids and clinical independent predictors) were performed. However, no statistic significant was found between the two models.

Lipids are crucial molecules involved in various biological processes, including membrane composition, energy metabolism, and signal transduction (15). Disruptions in lipid metabolism have been associated with tumorigenesis, indicating a potential utility for lipidomic profiles in identifying cancer biomarkers. The field of lipidomics, by delineating lipid profiles, offers insights into cancer’s metabolic alterations and facilitates biomarker identification (16). It has been effectively utilized in identifying novel biomarkers and characterizing lipid metabolic pathways in cancers such as lung, prostate, liver, and colorectal cancers (17). Previous research has highlighted the link between dysregulated lipid metabolism and the 5-year survival rate in lung cancer (18).

Previous studies showed that significant alterations in various lipid classes in lung cancer, including sphingomyelin (SM), ceramide (Cer), phosphatidylserine (PS), cholesterol ester (ChE), phosphatidylethanolamine (PE), phosphatidylcholines (PC), phosphatidylglycerol (PG), and fatty acids (FA) (19–21). However, until now, no serum lipidomic biomarkers have been identified for predicting response to chemo-immunotherapy in advanced NSCLC patients. Our findings contribute to the growing evidence of altered lipid metabolism in cancer, specifically identifying PC(16:0/18:2), PC(16:0/18:1), PC(16:0/18:0), CE(20:1), PC(14:0/18:1), and TAG56:7-FA18:2 as significant predictors. Phosphatidylcholines (PCs) are essential components of cell membranes and are involved in cell signaling and apoptotic pathways (19, 20). The specific PCs may reflect the altered cell membrane dynamics in cancer cells, affecting processes like cell proliferation, migration, and interaction with the immune system. Our study postulates that these PCs could influence the tumor microenvironment and thereby impact the response to chemo-immunotherapy.

Fatty acid metabolism is often disrupted in cancer cells. Previous studies have suggested that FAs can modulate cancer cell proliferation (22). Metabolic remodeling of FAs is also observed in inflammation and various cancers (23). For the triacylglycerol TAG56:7-FA18:2, our results suggest a potential link with the energy balance in cancer cells. Cancer cells often exhibit altered energy metabolism, and the dysregulation of triacylglycerols could reflect changes in how cancer cells utilize and store energy, potentially affecting their growth and survival under the metabolic stress induced by chemo-immunotherapy. The potential implications of our findings on treatment response are also explored. For example, the differential expression of TAG56:7-FA18:2 might influence the tumor’s metabolic adaptation to the cytotoxic environment created by chemo-immunotherapy, which could be leveraged to predict or even enhance treatment efficacy.

The role of cholesterol esters, such as CE(18:2), is known in atherosclerosis. However, their involvement in lung cancer tumorigenesis remains unclear (24). Our study suggested that CE(20:1) might play a role in chemo-immunotherapy response in NSCLC patients. This could be due to the involvement of cholesterol esters in the formation of lipid rafts, which are known to mediate signal transduction involved in cell proliferation and immune response modulation. Moreover, increased levels of PC and PE have been associated with NSCLC status (25, 26). We propose that the observed upregulation of certain PCs in our study might be indicative of an increased demand for membrane synthesis in rapidly proliferating tumor cells, while alterations in cholesterol esters like CE(20:1) might reflect changes in lipid raft composition, influencing signaling pathways that regulate cell proliferation and apoptosis (27, 28).

Additionally, we explored the potential correlation between lipidomic alterations and disease progression. Our results suggest that the dysregulation of specific lipid species may contribute to the modulation of the tumor microenvironment, affecting tumor growth, immune evasion, and response to immunotherapy. These findings not only advance our understanding of NSCLC biology but also propose a framework for employing lipidomic profiles in developing non-invasive, predictive tools for chemo-immunotherapy efficacy. This approach could significantly impact personalized treatment strategies, aligning with the broader goals of precision medicine in oncology.

However, this study had several limitations. The dynamic and sensitive nature of lipids necessitates further validation of our results for consistency and reproducibility. Additionally, larger-scale, multi-center, and prospective studies are required to provide more reliable evidence for clinical application.

## 5 Conclusions

Our study underscores the potential of lipidomic profiling as a highly accurate method for predicting chemo-immunotherapy responses in NSCLC. By offering a path towards personalized medicine, lipidomics could enhance therapeutic strategies for patients with NSCLC, marking a significant step forward in cancer treatment and management.

## Data availability statement

The raw data supporting the conclusions of this article will be made available by the authors, without undue reservation.

## Ethics statement

The studies involving humans were approved by Institutional Review Board of Jinshan Hospital. The studies were conducted in accordance with the local legislation and institutional requirements. The participants provided their written informed consent to participate in this study.

## Author contributions

HJ: Conceptualization, Writing – original draft. XL: Data curation, Formal analysis, Writing – original draft. YY: Data curation, Formal analysis, Writing – original draft. RQ: Conceptualization, Formal analysis, Funding acquisition, Writing – original draft.
