# Supplementary material for: Plasma lipidomics profiling in predicting the chemo-immunotherapy response in advanced non-small cell lung cancer
Source: Front Oncol. 2024 Jul 8;14:1348164. doi: 10.3389/fonc.2024.1348164 (PMC11260645; doi:10.3389/fonc.2024.1348164)
Supplement: Supplementary file 1 [file DataSheet_1.docx]

Supplementary Material

## Supplementary Tables

**Supplementary Table 1.** Multivariate logistic regression analysis of the clinical independent predictors for responders of chemo-immunotherapy.

| Coefficients | Estimate | Std. Error | t value | P |
| --- | --- | --- | --- | --- |
| (Intercept) | -0.4989 | 0.4067 | -1.2270 | 0.2249 |
| Gender | -0.0268 | 0.1320 | -0.2030 | 0.8400 |
| Age | 0.0124 | 0.0062 | 2.0040 | 0.0501 |
| Tumor location | -0.0087 | 0.1460 | -0.0600 | 0.9525 |
| Lung metastases | 0.2183 | 0.1332 | 1.6390 | 0.1066 |
| Brain metastases | -0.1174 | 0.1373 | -0.8550 | 0.3961 |
| Bone metastases | 0.1392 | 0.1310 | 1.0620 | 0.2926 |
| Liver metastases | 0.3388 | 0.1508 | 2.2470 | 0.0285* |
| Other metastases | 0.0370 | 0.2650 | 0.1400 | 0.8895 |
| Pathological subtype | 0.0270 | 0.0866 | 0.3110 | 0.7566 |

*, P < 0.05

## Supplementary Figures


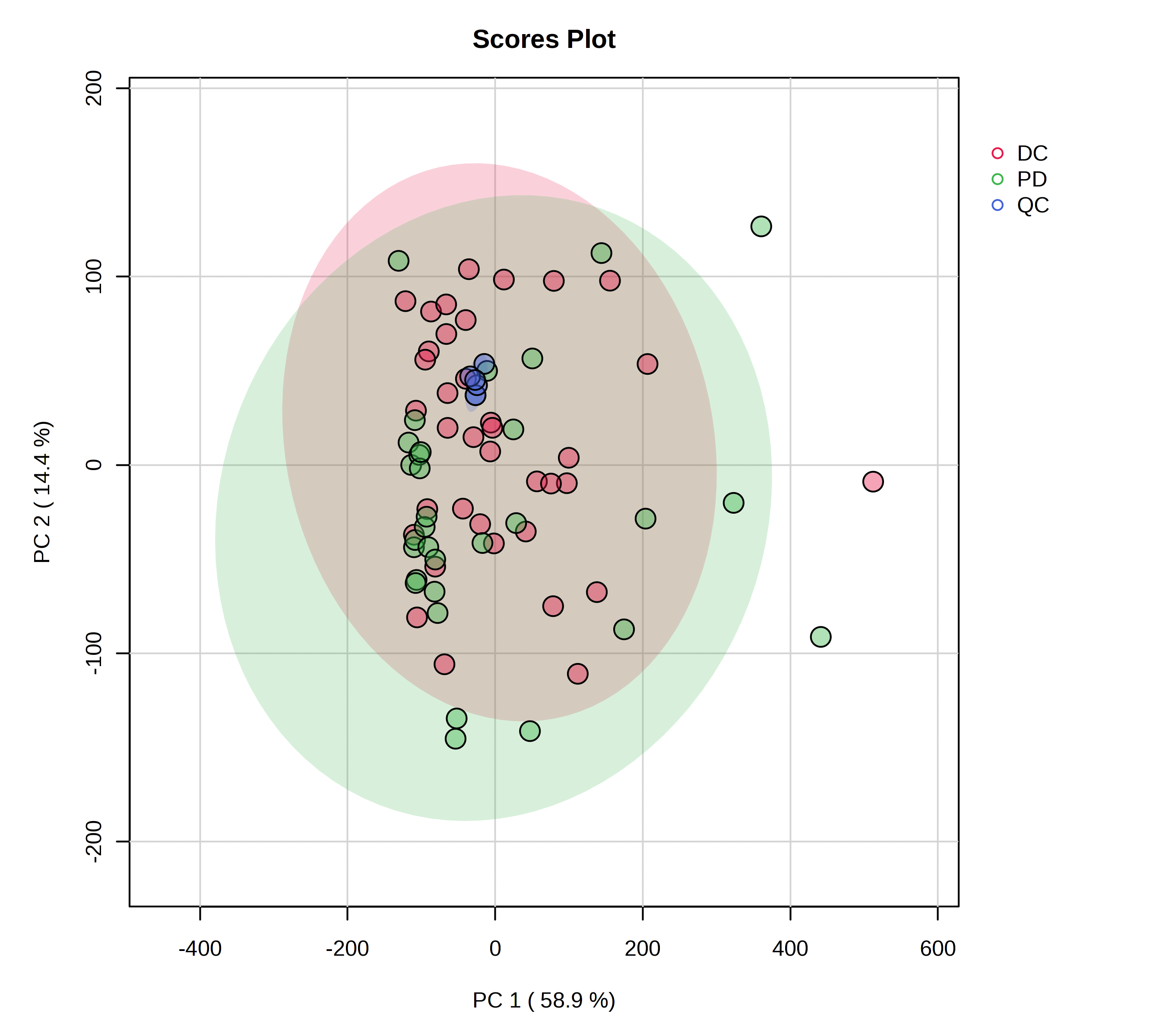


**Supplementary Figure 1.** Principal component analysis (PCA) plot of the lipids with QC samples.
